# Isolated primary cold abscess of the sternum: a case report

**DOI:** 10.1186/s13256-019-2210-9

**Published:** 2019-08-25

**Authors:** Lovenish Bains, Pawan Lal, Tirlok Chand, Kamal Kishore Gautam, Mohd Yasir Beg, Pritesh Kumar

**Affiliations:** Department of Surgery, Maulana Azad Medical College, New Delhi, India

**Keywords:** Tuberculosis, Sternum, Osteomyelitis, Cold abscess, Anti-tubercular therapy

## Abstract

**Background:**

Musculoskeletal tuberculosis forms 10–25% of extrapulmonary tuberculosis which mainly involves the spine or weight-bearing joints. Tuberculous involvement of the sternum is a rare clinical entity even in countries where tuberculosis has high prevalence. Primary tuberculous sternal osteomyelitis accounts for approximately 0.3% of all types of tubercular osteomyelitis and the probable source appears to be extension from paratracheal or hilar lymph nodes. Despite tuberculosis being a common disease in endemic countries and worldwide, a thorough literature search of the PubMed database for keywords “primary tuberculosis of sternum” and “primary tuberculous osteomyelitis of sternum” yielded 30 and 22 articles, respectively.

**Case presentation:**

We present an unusual case of a large dumb-bell-shaped cold abscess arising due to infection of the sternum. A 23-year-old immunocompetent Asian woman presented with a gradually progressing painless swelling on anterior chest wall for the last 5 months. She had a large visible swelling on anterior chest wall which was 12.5 cm in diameter, soft, non-tender, temperature was not raised, and fluctuant. Magnetic resonance imaging showed a large dumb-bell-shaped hyperintense collection in upper anterior chest wall with marrow edema and cortical irregularity in left side of manubrium. Pus was positive for nucleic acid testing (cartridge-based nucleic acid amplification test) for *Mycobacterium tuberculosis* and later culture was also positive. She was started on anti-tubercular therapy and aspirated twice. Currently, she has completed 6 months of therapy and the swelling has now disappeared.

**Discussion:**

Swelling, pain localized to sternum, or ulceration of the skin with discharging sinus along with or without constitutional symptoms are the usual presentation. A high element of suspicion is needed for early diagnosis and treatment to prevent its complications. Sternal mycobacterial infections are categorized as primary, secondary, and/or acquired postoperatively. Although radiological investigations aid in diagnosis, the diagnosis is established by positive culture or histopathological examination. Anti-tubercular therapy is the mainstay of treatment with standard four-drug regimen for 6–9 months. Surgical drainage of the abscess should be considered only if it does not resolve by aspiration and anti-tubercular therapy.

## Introduction

Skeletal tuberculosis (TB) accounts for 1–4% of patients with mycobacterial infection [1]. Any bone can be a site for TB, but sternum involvement is very uncommon. Primary sternal TB without pulmonary involvement is even more uncommon. Despite TB being a common disease in endemic countries and worldwide, a thorough literature search of the PubMed database for keywords “primary tuberculosis of sternum” and “primary tuberculous osteomyelitis of sternum” yielded 30 and 22 articles, respectively. Primary tuberculous sternal osteomyelitis accounts for approximately 0.3% of all types of tubercular osteomyelitis and the probable source appears to be extension from paratracheal or hilar lymph nodes [2]. This pattern of disease has been scarcely reported even from the endemic countries with high disease burden [3–5]. We present our experience of a young woman with large dumb-bell-shaped collection originating from the sternum as an isolated primary cold abscess of the sternum. This type of clinical presentation needs a high amount of suspicion for diagnosis and management.

## Case presentation

**Fig. 1:**
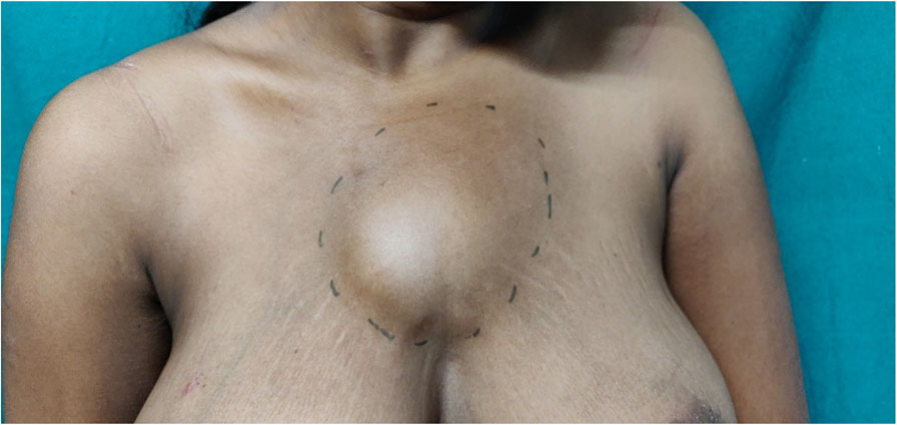
Large fluctuant anterior chest wall swelling

**Fig. 2:**
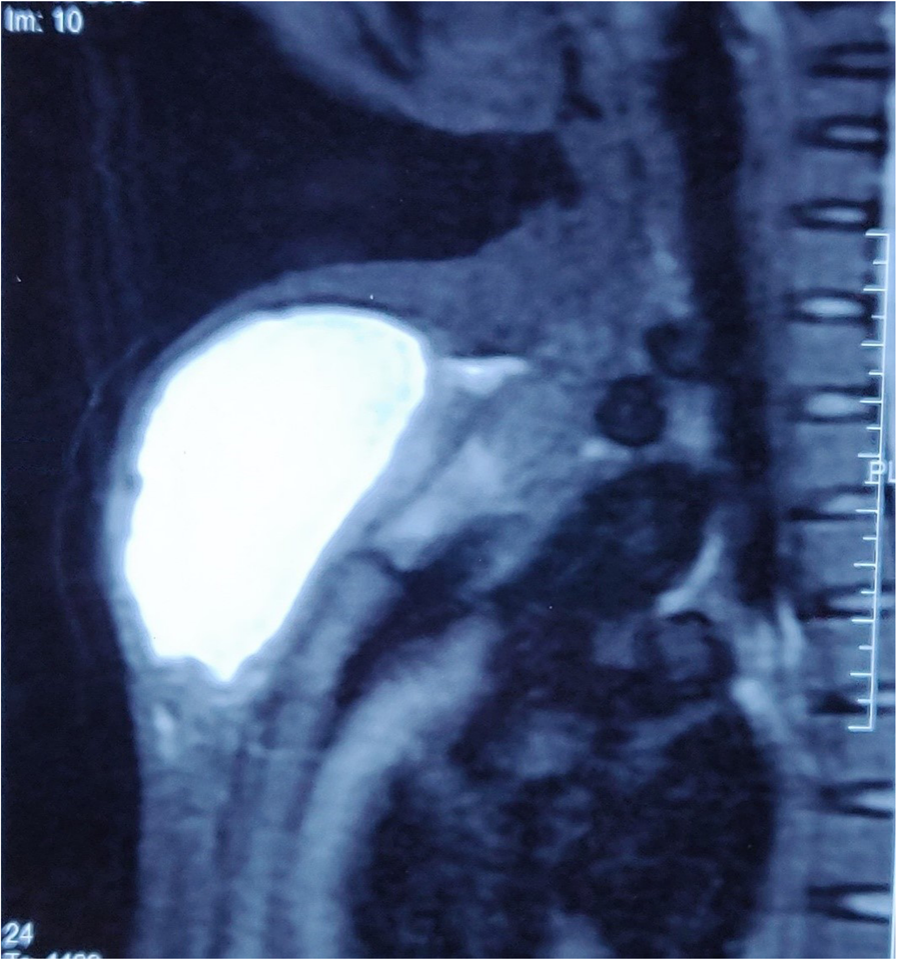
Magnetic resonance imaging (sagittal plane) showing the collection anterior to sternum

**Fig. 3:**
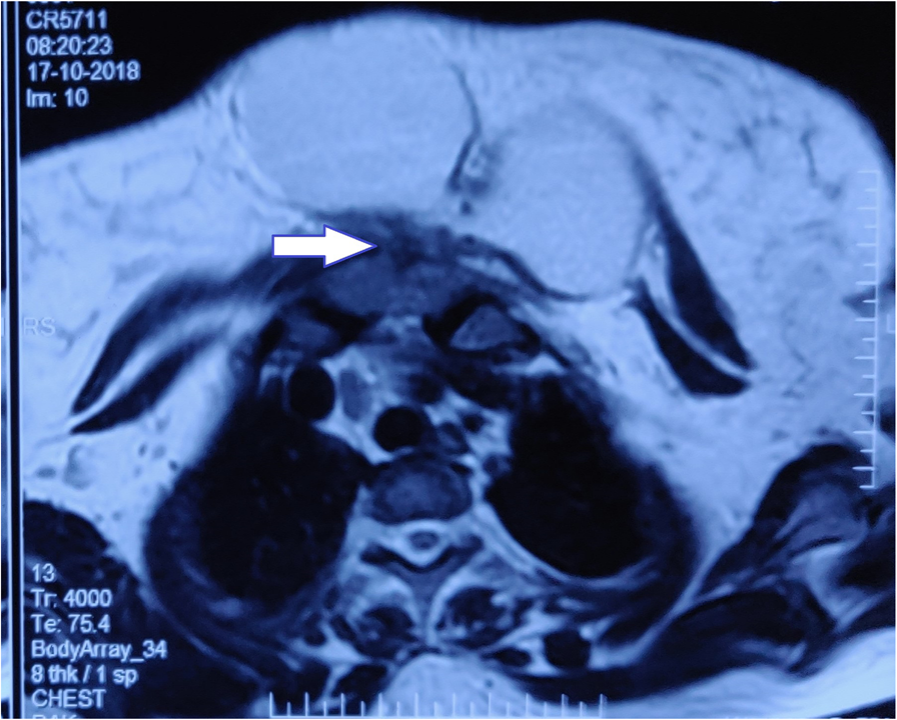
Cortical breach (*arrow*) in left side of manubrium

**Fig. 4:**
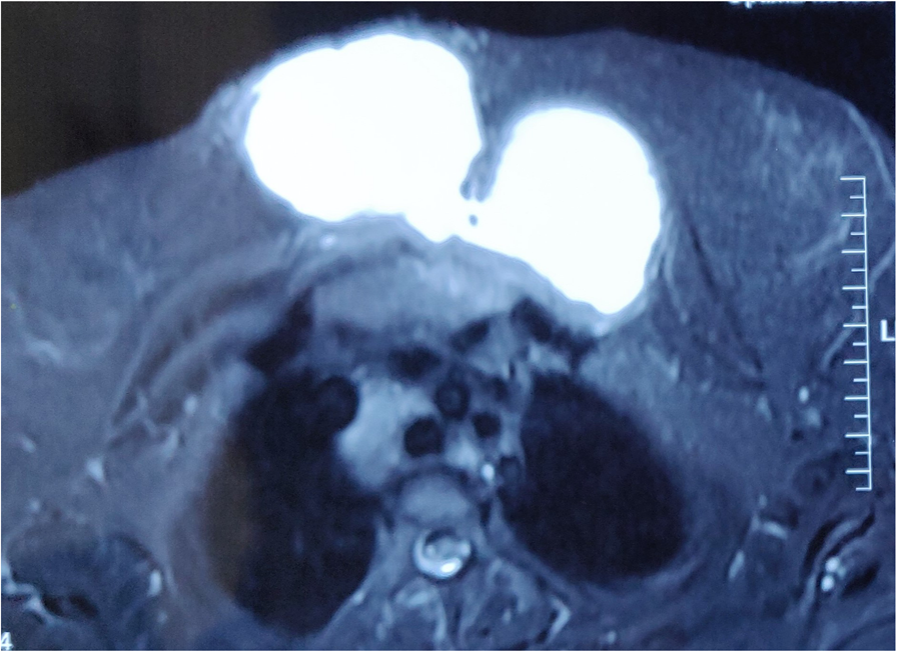
Magnetic resonance imaging (transverse plane) showing two large communicating collections

A 23-year-old woman of Asian descent (Caucasian) presented with a gradually progressing painless swelling on anterior chest wall for the past 5 months, with rapid increase in size in the last 1 month associated with increasing discomfort. She had no significant past history and was immunocompetent. There was no history of contact with Koch’s infection or in her family. There was no history of trauma to anterior chest wall or any surgery in the vicinity. She is a housewife and belongs to lower middle socioeconomic class as per modified Kuppuswamy scale. She had no addiction of any kind and received a 1-week course of antibiotics (amoxicillin and clavulanate) before presenting to us. Her Bacillus Calmette–Guérin (BCG) vaccination status was unconfirmed. She was of average build, afebrile, pulse rate 76/minute, respiratory rate 13/minute, and blood pressure 120/84 mmHg at presentation. There was a large visible swelling on anterior chest wall that was 12.5 cm in diameter, soft, non-tender, temperature was not raised, and fluctuant (Fig. 1). The overlying skin was normal and there was no other significant finding. There was no cervical lymphadenopathy. Chest and abdomen examinations and neurological assessment were essentially normal. Ultrasonography revealed a hypoechoic collection with approximately 180 cc contents. Her laboratory investigations were hemoglobin 9.1 g%, total leukocyte count of 8600 with 64% polymorphs and 35% lymphocytes, and erythrocyte sedimentation rate (ESR) 26 mm. Liver functions and renal functions were within normal range. A chest X-ray was also normal. Magnetic resonance imaging (MRI) showed a hyperintense collection in upper chest wall anterior to sternum measuring approximately 120 × 68 × 49 mm in size (Fig. 2) with marrow edema and cortical irregularity in left side of manubrium (Fig. 3) and another collection in vicinity which was communicating with primary swelling (Fig. 4).

**Fig. 5:**
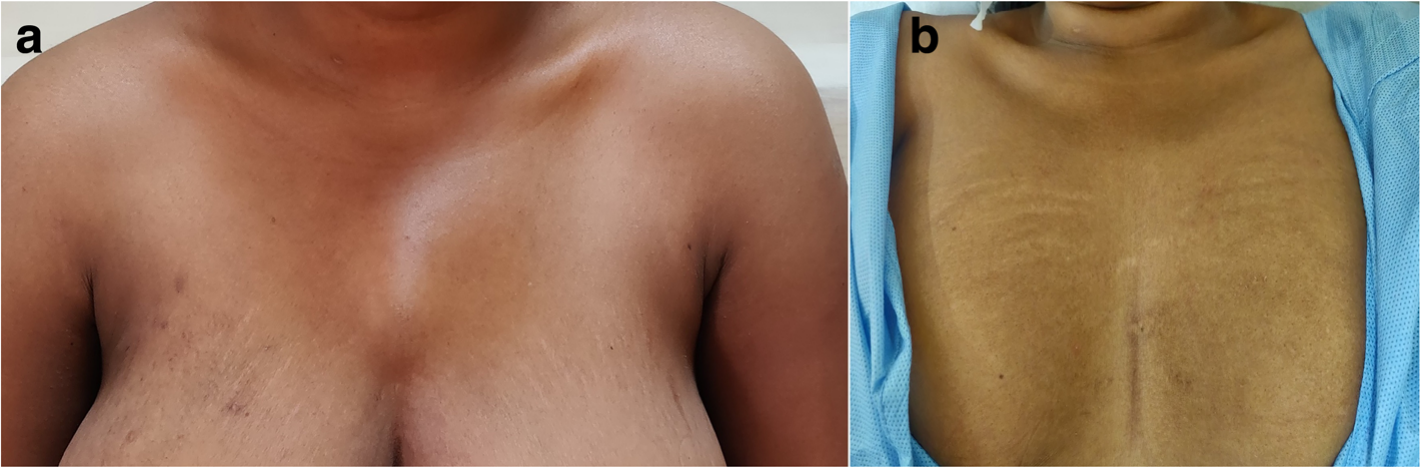
**a** Swelling decreased in size after anti-tubercular therapy and aspiration at 7 weeks. **b** Complete resolution after 6 months of anti-tubercular therapy

Few enlarged paratracheal lymph nodes were present. The pus was aspirated and subjected to investigations. The pus was negative for acid-fast bacilli (AFB) but nucleic acid testing by cartridge-based nucleic acid amplification test (CB-NAAT) for *Mycobacterium tuberculosis* was positive and susceptible to rifampicin. The pus culture showed no growth after 72 hours. A Mantoux test read at 48 hours was 4 mm; sputum examination and culture were negative for AFB. Her weight was 55 kg and she was started on daily dose anti-tubercular therapy (ATT) with four drugs, which were isoniazid (300 mg), rifampicin (450 mg), pyrazinamide (1200 mg), and ethambutol (800 mg), for 2 months (2HRZE) followed by 4 months of isoniazid and rifampicin (4HR). She needed two aspirations over 5 weeks initially. The culture became positive for *M*. *tuberculosis*. After 7 weeks of therapy, the swelling decreased considerably (Fig. 5a). Currently, she has completed 6 months of therapy and the swelling has now disappeared (Fig. 5b).

## Discussion

TB can disseminate to almost every organ after a primary infection or reactivation of latent foci. As per the Global TB report 2018, 10 million new cases occurred in 2017 and ten countries accounted for 80% of the cases with the top three being India (26%), Indonesia (11%), and Nigeria (9%) [6]. This implies that India accounts for about a quarter of the world’s cases of TB [6]. The rate of extrapulmonary TB (EPTB) worldwide has become 10–15% with young patients, females, and people from Africa or Asia seeming to have a higher risk [5]. Out of all cases of EPTB, 10–25% have musculoskeletal TB with the most common affected site being the spine (50–69%), followed by the hip, knee, and ankle/foot (10–13% each) [7].

Approximately 60 to 80% of cases of skeletal TB involve the spine or weight-bearing joints, while the sternum is involved in approximately 1% of cases [7–9]. TB of the sternum is a rare form of flat bone TB that may occur in isolation or in association with pleuropulmonary or lymph node involvement. Isolated primary cold abscess of the sternum becomes a diagnostic puzzle especially if pulmonary TB is not associated. Most of the cases are an extension from a primary focus in the chest and present with ulceration, discharging sinus, or a swelling with constitutional symptoms, whereas a large cold abscess without constitutional symptoms was present in our case.

Sternal mycobacterial infections have been categorized into three types: primary (67.3%), secondary (20.8%), and acquired postoperatively (11.9%) [5]. Tuli and Sinha reported 14 cases of sternal TB in a series of 980 cases of osteoarticular TB (1.5%) [3]. It mainly involves young males with a mean age of 36 years (range 11 to 59 years), although cases have been found in the pediatric population too [10]. The incidence in males is 65% whereas in females it is 34% [5]. Eyer *et al.* reviewed 27 articles reporting 32 cases from 1966 to 2013 and found that primary sternal TB was more common in men (76%) and occurred at a relatively young age [11].

The reactivation of latent loci formed during hematogenous or lymphatic dissemination of primary TB is the primary cause for tubercular sternal osteomyelitis. Direct extension from contiguous mediastinal lymph nodes or infection of retrosternal lymph nodes that erode into the sternum over time are other mechanisms [5, 9, 10, 12, 13]. Thoracic TB disease most commonly involves the shafts of the ribs or the costovertebral or costochondral junctions whereas lesions of the sternum when found are more common in the manubrium (almost 70%) than of the body [5]. Yuan found that isolated sternal TB was observed in 60.4% patients, sternal TB with peristernal tissue invasions (muscles, cartilages, and joints) in 20.1% patients, and sternal TB with concurrent TB of other organs in 19.5% patients [5].

The disease often gets detected late due to nonspecific symptoms and slow insidious course. The mean duration of symptoms prior to diagnosis was 6.3 months [11]. The clinical presentation of the disease is variable. Swelling and pain localized to the sternum are the most common symptoms reported. Others present with ulceration of the skin or discharging sinus. Constitutional symptoms are less commonly seen, but include malaise, fever, night sweats, or weight loss [2–5, 10–12, 14]. In our patient, constitutional symptoms were absent and she had a painless swelling to begin with, which became uncomfortable in the last month due to rapid progression in size. A workup was suggestive of primary sternal TB osteomyelitis and cold abscess.

Blood investigations are essentially normal in most cases apart from raised ESR. Chest radiographs are normal in approximately 70% of these cases, and approximately 40% have evidence of TB in sites other than the sternum, with the lymphatic system being the most common. More than 81% of cases of sternal TB osteomyelitis have an abnormal tuberculin skin test result [4, 10, 11, 13, 15].

According to a study done by Vijay *et al.*, radiological signs may not be present initially at the time of presentation, and symptoms, abscesses, or sinuses may be present long before imaging modalities detect them [16]. Plain radiographs are often normal but radiographic techniques like computed tomography (CT) and MRI are more valuable for localization and detection of bone destruction and soft tissue abnormalities. The common features on CT of sternoclavicular TB include bone and cartilage destruction, soft tissue masses crossing fascial planes with rim (abscess) and diffuse enhancement (granulation tissue), calcifications, and underlying pleuroparenchymal tubercular involvement [17–19]. MRI delineates abscesses better in the soft tissues, and highlights bone marrow involvement [5, 17–19]. Atasoy *et al.* demonstrated the role of MRI for early detection of marrow and soft tissue involvement in sternal TB due to the high contrast resolution of MRI [20]. Early changes of cellulitis (seen as replacement of subcutaneous fat signal on T1-weighted images with edema and enhancement) and myositis (showing hyperintensities of the involved muscles on T2-weighted images with their enlargement) are also frequently seen. Late changes are osteomyelitis, joint effusions, and bone destruction. Sinus tract formation, which appears as linear high signal intensity (SI) on T2-weighted images with marginal “tram-track enhancement”, may also be seen [17]. Ultrasound is of limited value in early stages but picks up abscesses, osteolytic sternal lesions, or rib lesions later [17].

A needle aspiration or excisional biopsy is mandatory for histopathological diagnosis of sternal osteomyelitis because radiological findings cannot differentiate the cause of osteomyelitis and sometimes may even appear neoplastic [21–23]. The diagnosis is usually confirmed by finding AFB and positive AFB cultures, and caseous necrosis and granuloma on histopathology [2, 3, 5, 10, 11, 13, 21]. The frequency of positive cultures is up to 75%. The newer tests like polymerase chain reaction (PCR) amplification and GeneXpert nucleic acid amplification test (NAAT) can also aid the diagnosis in cases of negative smear or culture.

A high index of suspicion is required for early diagnosis and prompt treatment that can prevent complications. ATT is the mainstay of treatment with standard four-drug regimen for 6–9 months. Cold abscess or collections can be aspirated. Khan *et al.* found that surgical intervention was only necessary if there was: a need for an open biopsy when needle aspiration is inconclusive; draining sinuses; debridement to promote early healing for markedly damaged or sequestrated bones or joint on radiographs, such as pectus excavatum; extensive mediastinal disease or worsening disease; signs of secondary infection or mediastinitis; or disease not responding to an effective course of ATT [19]. The surgical options are needed only for persistent draining sinus and bone destruction which comprises thorough debridement followed by pectoralis major, rectus abdominis, latissimus dorsi, or omental flap closure, with or without chest wall reconstruction or vacuum-assisted closure [19, 24, 25]. The prognosis of the patients is usually good with treatment.

## Conclusion

Isolated primary sternal osteomyelitis due to *M*. *tuberculosis* is still rare despite the high prevalence of TB in endemic countries. Tubercular involvement of the sternum can occur with various presentations and can involve any age group. It needs a high index of suspicion as diagnosis is usually delayed. A CT scan and MRI provide essential clues but confirmation is by culture or histopathological examination. ATT remains the mainstay of treatment. Surgical drainage of the abscess should be considered only if it does not resolve by aspiration and ATT.

## Data Availability

Not available.

## Abbreviations

AFB: Acid-fast bacilli
ATT: Anti-tubercular therapy
BCG: Bacillus Calmette–Guérin
CB-NAAT: Cartridge-based nucleic acid amplification test
CT: Computed tomography
EPTB: Extrapulmonary tuberculosis
ESR: Erythrocyte sedimentation rate
*M. tuberculosis*: *Mycobacterium tuberculosis*
MRI: Magnetic resonance imaging
NAAT: Nucleic acid amplification test
PCR: Polymerase chain reaction
SI: Signal intensity
TB: Tuberculosis
